# Supplementary material for: Behavior of Piglets in an Observation Arena before and after Surgical Castration with Local Anesthesia
Source: Animals (Basel). 2023 Feb 2;13(3):529. doi: 10.3390/ani13030529 (PMC9913414; doi:10.3390/ani13030529)
Supplement: Supplementary file 1 [file animals-13-00529-s001.zip › animals-2184420-supplementary.pdf]

Table S1. Termination criteria during observation of piglets in observation arena; piglets that showed these behaviors were immediately removed from the observation arena and returned to their home pens.

**Termination criterion**	**Definition**
Nervousness	Animal appears scared, shows trembling or jumpiness
Pivoting	Animal is continuously in motion and moves around in circles
Escape attempts	Piglet is rising on hind legs more than once or attempts to jump out of the observation arena
High-frequency vocalization	Piglet makes repeated high-frequency calls
Freezing	Piglet stops all movement and doesn't react to outside stimuli
